# A Novel Monoclonal Antibody Targeting the A29 Protein of Monkeypox Virus and Its Application in Immunoassay

**DOI:** 10.3390/antib15030045

**Published:** 2026-05-29

**Authors:** Nan Jia, Weixiao Wang, Guangwei Zhao, Danfei Meng, Liyuan Zheng, Jinhua Dong

**Affiliations:** 1 School of Life Science and Technology, Shandong Second Medical University, Weifang 261053, China; 2 School of Rehabilitation Sciences and Engineering, University of Health and Rehabilitation Sciences, Qingdao 266113, China; 3 School of Basic Medical Sciences, Shandong University, Ji’nan 250014, China; 4 School of Basic Medicine, Qingdao University, Qingdao 266071, China; 5 Institute of Integrated Research, Institute of Science Tokyo, Yokohama 226-8503, Japan

**Keywords:** monkeypox virus, A29, monoclonal antibody, immunoassay

## Abstract

Background: The monkeypox virus (MPXV) has attracted considerable global attention due to its potential to cause widespread outbreaks, necessitating the development of rapid and accurate diagnostic methods of significant clinical importance. A29, a key envelope protein of MPXV, represents a promising diagnostic target. Methods: A novel monoclonal antibody, D10, was isolated from the human Tomlinson I+J phage display library by biopanning against the recombinant A29 protein. The D10 Fab fragment was expressed and purified, and its binding affinity was characterized by biolayer interferometry. Molecular docking was performed to predict potential interacting residues. Specificity and detection performance were evaluated by direct and competitive enzyme-linked immunosorbent assay (ELISA). Results: D10 possesses a unique complementarity-determining region sequence and exhibits strong binding affinity toward the A29 protein. Structural modeling analysis suggested potential interacting residues of A29, including Gln67, Arg74, Asn75, Arg81, and Asn84, which may primarily interact with Ser10, Thr5, Gly49, Gly47, and Glu97 in the heavy chain of D10. The binding affinity, determined by biolayer interferometry, showed a dissociation equilibrium constant of 6.44 nM, indicating strong binding capability. Furthermore, competitive ELISA demonstrated that D10 binds selectively to the A29 protein, with a half-maximal inhibitory concentration of 1.88 μg/mL and a limit of detection of 0.12 μg/mL. Conclusions: Overall, this monoclonal antibody provides a valuable tool for the immunological detection of MPXV and holds potential for future clinical diagnostic applications.

## 1. Introduction

Monkeypox virus (MPXV) is a member of the Orthopoxvirus genus within the Poxviridae family [1]. In recent years, particularly following the COVID-19 pandemic, MPXV has emerged as a viral pathogen of increasing global concern due to its outbreak potential [2,3,4]. A29, an intracellular mature virion (IMV) envelope protein of MPXV, mediates viral attachment to host cells via interactions with heparin and heparan sulfate [5]. As a major antigen capable of eliciting host immune responses, A29 also represents an important target for antibody-based detection. A29, a key envelope protein of MPXV, exhibits low sequence homology to related orthopoxvirus proteins and has been reported as a specific diagnostic target [5,6]. Therefore, the development of detection strategies targeting A29 is of considerable importance for early diagnosis, disease monitoring, and therapeutic evaluation of monkeypox infection. Currently, the diagnosis of MPXV primarily relies on polymerase chain reaction (PCR)-based detection of viral nucleic acids [7,8]. Although PCR offers high specificity, it requires specialized instrumentation and trained personnel, which limits its applicability in resource-limited settings and point-of-care scenarios. In contrast, immunoassays based on antigen–antibody interactions provide a more accessible and operationally simple alternative, with advantages including high sensitivity and rapid detection [9,10,11]. Phage display technology offers an alternative approach for antibody generation that does not require animal immunization, enables rapid in vitro selection, and allows direct isolation of fully human antibodies. These features are particularly valuable for developing diagnostic reagents against emerging pathogens such as MPXV [12,13]. Accordingly, there is a critical need to develop monoclonal antibodies with high specificity and strong affinity. In this study, a monoclonal antibody targeting the A29 protein was successfully isolated using phage display technology, and its binding affinity and specificity were systematically characterized by enzyme-linked immunosorbent assay (ELISA), biolayer interferometry (BLI) and molecular docking.

## 2. Materials and Methods

### 2.1. Materials

Plasmids for the prokaryotic expression of *A29L* were synthesized by Ruibo Xingke Biotechnology Co., Ltd. (Beijing, China). The phage display library Tomlinson I+J, a human synthetic library constructed by cloning synthetic human V-gene repertoires into the phagemid vector pIT2, was obtained from Source BioScience, Nottingham, UK. Helper phage M13KO7 and restriction enzymes were obtained from New England BioLabs (Beijing, China). *Escherichia coli* Rosetta (DE3) competent cells for A29 protein expression were purchased from Agilent Technologies (La Jolla, CA, USA). *E. coli* SHuffle T7 cells used for antigen-binding fragment (Fab) expression were obtained from New England BioLabs (Beijing, China). A29 protein and Fab purification were performed under native conditions using Ni-TED Sepharose 6HP affinity chromatography resin (Sangon Biotech, Shanghai, China). Recombinant A29 protein expressed in HEK293 cells was purchased from Nanjing Okay Biotechnology Co., Ltd. (Nanjing, China). Recombinant A27, H3 and B6R proteins were purchased from Sino Biological Inc. (Beijing, China). The mouse monoclonal antibody targeting MPXV A29 was purchased from Sino Biological Inc. (Beijing, China). NHS-biotin for labeling of A29 protein was purchased from Sangon Biotech (Shanghai, China).

### 2.2. Preparation of A29 Protein

The *A29L* gene sequence of monkeypox virus was obtained from the NCBI GenBank database under accession number NC_003310.1. The coding sequence for the mature A29 protein corresponding to amino acid residues Met 1 to Glu 110 was synthesized and cloned into the pET22b vector. The recombinant plasmid pET22b-A29L was transformed into *E. coli* Rosetta (DE3) competent cells and plated on selective agar plates. Plates were incubated at 37 °C for 12 h. Positive colonies were selected by PCR and cultured in Luria–Bertani (LB) medium containing ampicillin, followed by induction with 1 mM isopropyl β-D-thiogalactoside (IPTG). After expression, A29 protein was purified by His-tag affinity chromatography. Protein purity was confirmed by using sodium dodecyl sulfate-polyacrylamide gel electrophoresis (SDS-PAGE). The antibody-binding activity of the purified A29 protein was assessed by an indirect ELISA. Briefly, 96 well plates were coated with 5 µg/mL A29 and bovine serum albumin (BSA) in phosphate-buffered saline (PBS) at 4 °C overnight. After blocking with PBS containing 5% skim milk (MPBS) at 23 °C for 2 h, the commercial mouse anti-A29 monoclonal antibody (1:2000 dilution) was added and incubated at 23 °C for 1 h. Following six washes with PBS containing 0.1% Tween-20 (Polyoxyethylene (20) sorbitan monolaurate; PBST), horseradish peroxidase (HRP) conjugated goat anti-mouse IgG (1:5000 dilution) was added and incubated for 1 h. After five additional washes, 3,3′,5,5′ -tetramethylbenzidine (TMB) substrate was added and incubated for 1 h. The reaction was stopped with 2M sulfuric acid, and the absorbance was measured at 450 nm with background subtraction at 630 nm using a microplate reader.

### 2.3. Panning of A29 Specific Monoclonal Antibody

A29 protein at concentration of 5 μg/mL was coated onto a 96-well plate and incubated overnight at 4 °C. After blocking with MPBS for 2 h at 23 °C, the plate was washed three times with PBST. The Tomlinson I+J phage display library (R0) was diluted to 10^10^ cfu(colony-forming unit)/mL and added to the A29-coated wells and incubated at 23 °C for 2 h. Unbound phages were removed by 10 washes with PBST, and bound phages were eluted with glycine-HCl buffer (pH 2.2), followed by immediate neutralization with Tris-HCl buffer (pH 7.4). The eluted phages were used to infect log-phase *E. coli* TG-1 (OD_600_ = 0.4) for 30 min. The bacterial cells were pelleted by centrifugation, resuspended in 2YT medium (16 g/L tryptone, 10 g/L yeast extract, 5 g/L NaCl, pH 7.2) containing 100 μg/mL ampicillin and 1% glucose (2YTAG), and cultured overnight for amplification. The following day, the culture was superinfected with helper phage M13KO7, and the medium was replaced with 2YTAK supplemented with 0.1% glucose (2YTAGK). The cells were then incubated at 30 °C with shaking for 20 h. Phage particles were harvested from the supernatant via PEG/NaCl (20% polyethylene glycol 6000/2.5 M NaCl) precipitation and resuspended in sterile PBS to generate the first-round enriched library (R1). After three iterative rounds of panning under the same conditions, the R2 and R3 libraries were sequentially enriched. Phage titers of R1, R2, and R3 were determined by infecting *E. coli* TG-1 with serially diluted phage libraries followed by colony counting on agar plates. Binding specificity of the R0-R3 libraries toward A29 was evaluated by ELISA using A29-coated wells and BSA-coated wells as a negative control. The enriched R3 library was further subjected to titration analysis. Ninety-six individual clones were randomly selected, cultured, and used for phage preparation. Phage supernatants were added to wells of microplate on which A29 or BSA had been previously coated and incubated. Clones showing specific binding to A29 were identified by ELISA. Eight positive clones were sequenced and analyzed against antibody gene databases. A novel monoclonal antibody was identified and designated as D10.

### 2.4. Expression, Purification, and Activity of Antigen-Binding Fragments

Based on the variable region sequences of the heavy (V_H_) and light (V_L_) chains of D10, specific primers were designed. V_H_ and V_L_ gene fragments were amplified by PCR using primer pairs AgeI-VH-F/XhoI-VH-R for V_H_ gene and SpeI-VL-F/HindIII-VL-R for V_L_ gene (Table 1). PCR products and the pUQ2GS vector were digested with corresponding restriction enzymes, purified, and ligated to construct the recombinant plasmid pUQ2GS-D10 Fab. The verified plasmid was transformed into *E. coli* SHuffle T7 competent cells for prokaryotic expression. Protein expression was induced with 0.5 mM IPTG at 16 °C for 16 h. The expressed Fab was purified from bacterial lysates using Ni-TED affinity chromatography resin under native conditions, and the purity and integrity of the purified D10 Fab were assessed by reducing SDS-PAGE. To validate the antigen-binding activity of D10 Fab, ELISA was performed using a unified protocol: 96-well plates were coated overnight at 4 °C with 5 μg/mL of *E. coli*-expressed A29, HEK293-expressed A29, and BSA diluted in PBS. After blocking with MPBS for 2 h at 23 °C, the plates were washed and then incubated separately with either D10 Fab (10 μg/mL) for 1 h at 23 °C. The plates were sequentially incubated with an anti-His tag mouse monoclonal antibody (1:2000 dilution) for 1 h at 23 °C, followed by HRP-conjugated goat anti-mouse IgG (1:4000 dilution) for 1 h at 23 °C. After each antibody incubation, plates were washed three times with PBST. Finally, the absorbance was measured at 450 nm with background subtraction at 630 nm using a microplate reader.

### 2.5. Affinity Measurement of D10 Fab by Biolayer Interferometry

A29 protein at 0.5 mg/mL was reacted with NHS-biotin at a molar ratio of 1:20 at 4 °C for 2 h. Excess biotin was removed by dialysis to obtain biotinylated A29, and labeling efficiency was verified by an ELISA. Briefly, 96-well plates were coated with 5 μg/mL of streptavidin (SA) in PBS (100 μL/well) overnight at 4 °C. After blocking with MPBS for 1 h at 23 °C, the wells were incubated with either biotinylated A29 and A29 without biotinylation (10 μg/mL) for 1 h at 23 °C. The commercial mouse monoclonal antibody against MPXV A29 (1:2000 dilution) was then added and incubated for 1 h at 23 °C. After washing six times with PBST, HRP-conjugated goat anti-mouse IgG (1:5000 dilution) was added and incubated for 1 h at 23 °C. Following additional washes, TMB substrate was added and incubated for 15 min at 23 °C in the dark. The reaction was stopped with 2 M sulfuric acid, and the absorbance was measured at 450 nm with background subtraction at 630 nm using a microplate reader. For affinity measurement, binding kinetics were measured using a Gator Prime instrument. SA biosensors were equilibrated in kinetic buffer (1× PBS, pH 7.4, containing 0.02% Tween-20 and 0.2% BSA) for 10 min and then loaded with 10 μg/mL biotinylated A29 until signal stabilization. D10 Fab was tested at concentrations of 200, 500, 1000, and 2000 nM. Association and dissociation were monitored for 200 s each. Sensors without antigen loading served as controls. The binding kinetics were fitted using a 1:1 Langmuir binding model (global fitting) implemented in the Gator Prime 2.18.7 software. Data were analyzed using Data Analyze 12.0 software to calculate the dissociation equilibrium constant (*K*_D_) value.

### 2.6. Molecular Docking

To investigate the interaction mechanism between D10 single-chain variable fragment(scFv) and the A29 protein, molecular docking simulations were performed. First, 3D structures of the antigen and antibody were predicted. The amino acid sequence of A29 was submitted to the ColabFold server (based on AlphaFold2) to generate five structural models. The quality of each model was evaluated based on the predicted local distance difference test (pLDDT) scores output by the server, and the model with the highest pLDDT score was selected as the antigen structure for subsequent docking. Meanwhile, the amino acid sequence of D10 scFv was submitted to the Swiss Model online server for homology modeling. By searching the Protein Data Bank (PDB) for known antibody structures with high sequence similarity as templates, a 3D model of D10 scFv was constructed. After structure preparation, molecular docking was carried out using the HDOCK online server. Following docking, the complex with the optimal docking score was selected and visualized using PyMOL 2.5.7 for further analysis.

### 2.7. Validation of Cross-Reactivity of D10

BSA, A29, A27, H3, and B6R were coated at 2 μg/mL in PBS and incubated overnight at 4 °C. The next day, the plates were blocked with MPBS for 2 h at 23 °C, followed by three washes with PBST. Then, phage-displayed D10 scFv was added to each well and incubated for 1 h at 23 °C. Afterward, HRP-conjugated anti-M13 antibody (diluted 1:5000) was added to each well and incubated for 1 h at 23 °C. Following an additional washing step, TMB substrate was added for color development. The reaction was terminated by adding sulfuric acid, and the absorbance was measured at the appropriate wavelength using a microplate reader.

### 2.8. Competitive ELISA for A29 Detection

A29 protein and BSA were diluted to 1 μg/mL in PBS and coated onto 96-well plates at 4 °C overnight. After coating, the plates were blocked with MPBS for 2 h at 23 °C to reduce non-specific binding. For specificity assessment, BSA-coated wells were incubated with phage-displayed D10 scFv (1 × 10^10^ cfu/mL) and served as negative controls. For competitive detection, A29-coated wells were incubated with phage-displayed antibodies pre-mixed with free A29 at concentrations of 0, 8, 40, 200, 1000, 5000, and 25,000 ng/mL. The mixtures were incubated at 23 °C for 1 h to allow competitive binding between soluble and immobilized antigen. Following incubation, the wells were washed thoroughly with PBST to remove unbound phages. Subsequently, HRP-conjugated anti-M13 antibody (1:5000 dilution) was added and incubated for 1 h at 23 °C. After an additional washing step, TMB substrate was added for color development. The reaction was terminated by the addition of sulfuric acid, and absorbance was measured at the appropriate wavelength using a microplate reader.

### 2.9. Data Analysis

The cross-reactivity of D10 with each tested protein was calculated using the following formula: (Absorbance of tested protein/Absorbance of A29 protein) × 100%.

For the competitive ELISA, the half maximal inhibitory concentration (IC_50_) value was determined by fitting the competitive inhibition curve to a four-parameter logistic (4PL) model using GraphPad Prism 9.0, and the limit of detection (LOD) was calculated as the concentration of free A29 corresponding to the mean absorbance of the zero-standard minus three times the standard deviation (mean − 3 × SD), followed by interpolation from the same 4PL standard curve.

## 3. Results

### 3.1. The Expression and Purification Effect of A29 Protein

**Figure 1:**
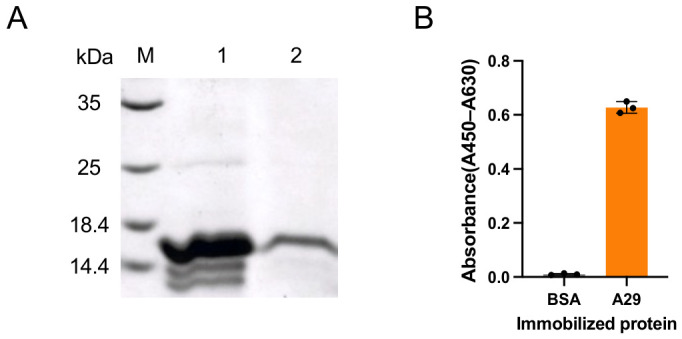
Expression and purification of recombinant A29 protein. (**A**) SDS–PAGE analysis of purified A29 protein under reducing conditions, followed by Coomassie Brilliant Blue staining. Lane M: Molecular weight markers for reference. Lanes 1 and 2 correspond to the first and second elution fractions collected using 250 mM imidazole, with 10 μL of each eluate loaded onto the gel. A distinct protein band was observed at approximately 15 kDa, consistent with the predicted molecular weight, indicating successful expression and high purity of the recombinant protein. (**B**) Evaluation of antigen-binding activity of purified A29 protein by an ELISA. The experimental group exhibited a significantly higher absorbance signal compared with the negative control, demonstrating that the purified A29 protein retained its antigenicity and was capable of specific antibody recognition.

The expression and purification of recombinant A29 protein were first evaluated by SDS–PAGE. As shown in Figure 1A, a prominent single band was observed at approximately 15 kDa, which is consistent with the predicted molecular weight of the A29 protein. The absence of significant non-specific bands or degradation products indicates that the recombinant protein was successfully expressed and purified with high purity under native conditions. These results demonstrate the effectiveness and reliability of the prokaryotic expression and purification system used in this study. To further assess the biological activity and antigenicity of the purified A29 protein, ELISA was performed. As illustrated in Figure 1B, the absorbance signal obtained from the experimental group was markedly higher than that of the negative control group. This significant difference indicates that the purified A29 protein retained its structural integrity and antigenic epitopes, enabling specific recognition by antibodies. Taken together, these results confirm that the recombinant A29 protein was successfully expressed with high purity and maintained its functional activity, thereby providing a reliable antigen for subsequent antibody panning and characterization experiments.

### 3.2. Panning and Sequence Analysis of Monoclonal Antibodies

**Figure 2:**
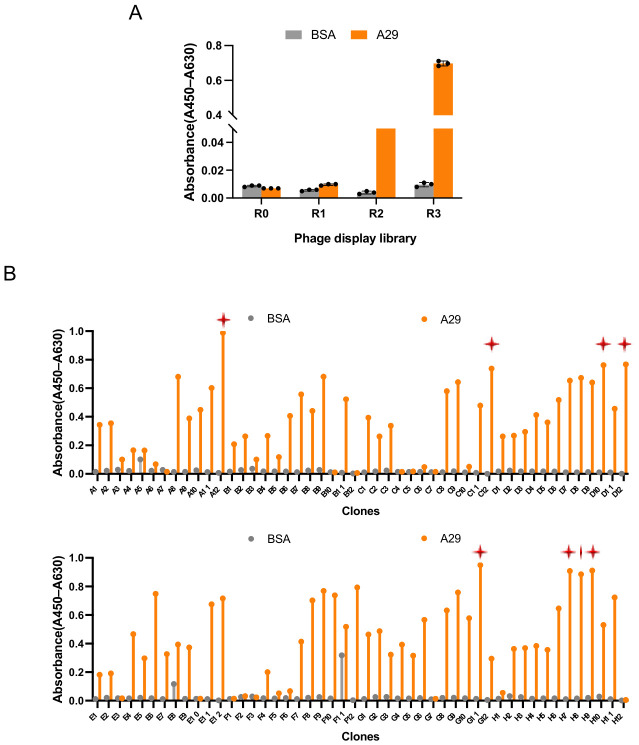
Panning and identification of A29-specific monoclonal antibodies. (**A**) Enrichment of A29-specific phage-displayed antibodies through successive rounds of biopanning. The binding activity of the phage library to immobilized A29 protein increased progressively with each round, while binding to BSA, used as a negative control, remained low, indicating effective and specific enrichment of target-binding clones. (**B**) Evaluation of antigen-binding activity of individual monoclonal phage clones by phage ELISA. Phage supernatants from selected clones were incubated with A29- or BSA-coated wells. 8 Clones exhibiting strong binding signals to A29 and negligible reactivity to BSA that are labeled with a red four-pointed star were selected, followed by plasmid extraction and gene sequencing.

The binding activity of the phage display libraries (R0, R1, R2 and R3) toward the A29 protein was markedly enhanced with the progressive increase in the number of biopanning rounds, as shown in Figure 2A. In contrast, binding to BSA, used as a negative control, remained consistently low across all rounds. This trend indicates a gradual and effective enrichment of phage clones specifically recognizing the A29 antigen, demonstrating the efficiency and selectivity of the biopanning process. To further evaluate the enrichment outcome, individual clones from the third-round library (R3) were subjected to ELISA-based panning. As presented in Figure 2B, a substantial proportion of monoclonal phage clones exhibited strong binding signals toward A29, whereas negligible reactivity was observed against BSA. These results confirm the successful isolation of A29-specific binders. Subsequently, eight representative positive clones, A12, C12, D10, D12, F12, H7, H8 and H9 were selected for DNA sequencing and sequence analysis. All eight sequenced clones displayed identical complementarity-determining region (CDR) sequences, indicating they are clonally related. The representative sequences of D10 are summarized in Table 2. Based on sequence comparison with entries in the IMGT database, no identical antibody sequence was identified, indicating that the selected clone represents a previously unreported antibody. This novel monoclonal antibody was designated as D10. Collectively, these findings demonstrate that phage display technology enabled the efficient enrichment and identification of a highly specific and previously uncharacterized monoclonal antibody targeting the MPXV A29 protein.

### 3.3. Characterization of D10 Fab: Antigen Binding and Affinity

**Figure 3:**
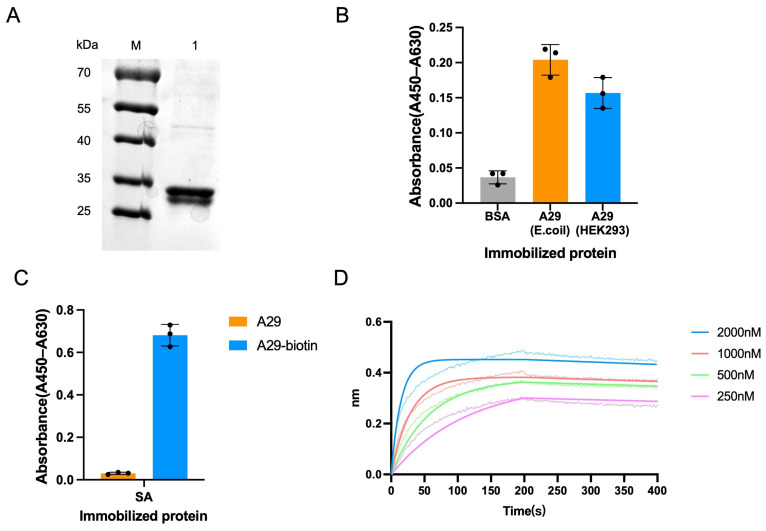
Expression, antigen-binding activity, and affinity characterization of the D10 Fab fragment. (**A**) SDS–PAGE analysis of purified D10 Fab under reducing conditions, followed by Coomassie Brilliant Blue staining. Lane M: Molecular weight markers for reference. Lane 1: purified D10 Fab. (**B**) Evaluation of the antigen-binding activity of purified D10 Fab by ELISA. Wells were coated with A29 protein derived from HEK293 cells (A29 (HEK293)), A29 protein derived from *E. coli* (A29 (*E.coli*)), and BSA (negative control). (**C**) Assessment of the antigenicity of biotinylated A29 protein by ELISA. Biotinylated A29 was captured on streptavidin (SA)-coated plates, whereas unlabeled A29, used as negative control, was not captured, leading to a low background signal. The signal ratio reflects the efficiency of the capture system. (**D**) Biolayer interferometry (BLI) sensorgrams showing the binding kinetics of D10 Fab to immobilized A29-biotin. The dashed lines represent the raw data, while the solid lines denote the fitted curves.

The expression and purification of the D10 Fab were first evaluated by SDS–PAGE under reducing conditions. As shown in Figure 3A, two distinct protein bands corresponding to the heavy chain and light chain were clearly observed, with apparent molecular weights consistent with their theoretical values. The absence of significant impurity bands indicates that the Fab was successfully expressed, correctly assembled, and purified to a high degree of homogeneity. The antigen-binding capability of D10 Fab was subsequently assessed by ELISA. As illustrated in Figure 3B, D10 Fab exhibited strong and specific binding to A29 proteins derived from both *E. coli* and HEK293 cells, with comparable levels, whereas negligible binding was observed to the control BSA. Importantly, the ability of D10 Fab to recognize the eukaryotic HEK293-expressed A29 protein demonstrates that it binds to the native, properly folded, authentic conformation of the A29 antigen. To enable affinity measurement by BLI, A29 protein was biotinylated. The functionality of the biotinylated antigen was verified by ELISA (Figure 3C), which demonstrated that the modification process did not compromise its binding capacity. This step ensured the reliability of subsequent kinetic measurements. Binding kinetics between D10 Fab and immobilized A29-biotin were then analyzed using a BLI-based assay. As shown in the sensorgram (Figure 3D), a clear association phase was observed upon exposure to the antibody, followed by a well-defined dissociation phase after transfer to buffer, indicating a stable and reversible interaction. The kinetic data were fitted using an appropriate binding model, and D10 Fab’s *K*_D_ value was calculated to be 6.44 nM (Table 3).

These results demonstrate that D10 Fab exhibits high-affinity binding to the A29 protein at the nanomolar level, supporting its potential application in sensitive immunodetection and diagnostic assay development.

### 3.4. Structural Analysis

**Figure 4:**
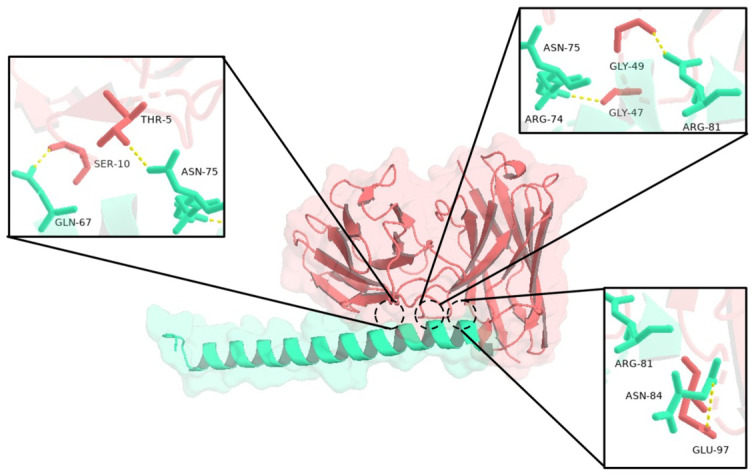
Predicted structural model of the D10 scFv–A29 complex. The 3D structure of the D10 scFv in complex with the A29 antigen was generated by molecular docking and visualized using PyMOL. The D10 scFv is shown in red, while the A29 protein is depicted in green. The predicted binding interface reveals close spatial complementarity between the antibody and antigen. Putative hydrogen bond interactions at the interface are indicated by yellow dashed lines, highlighting residues that may be involved in binding. These predicted interactions are primarily located within the V_H_ region of D10, near the CDRs, suggesting a potential role in antigen recognition and high-affinity binding.

A structural model of the D10 scFv–A29 complex was generated using the HDOCK online docking platform. Among the top 10 predicted models, Model 1 was selected for further analysis based on its highest docking score and favorable interface complementarity. The key metrics for Model 1 are as follows: docking score = −188.02, confidence score = 0.6814, and ligand RMSD = 97.86 Å. The resulting complex was subsequently visualized and analyzed with PyMOL 2.5.7 software (Figure 4). The docking model exhibited a well-defined binding interface between the antibody and antigen, allowing detailed characterization of the predicted intermolecular interactions. Structural analysis suggested that the interaction between D10 scFv and A29 is predominantly mediated by hydrogen bonding, supplemented by local spatial complementarity at the binding interface. Several residues on the A29 protein were predicted to contribute to the interaction, including Gln67, Arg74, Asn75, Arg81, and Asn84. These residues may form hydrogen bonds and close contacts primarily with residues Ser10, Thr5, Gly49, Gly47, and Glu97 located in the variable heavy (VH) region of D10 scFv. Notably, Arg74 and Arg81 of A29 may establish direct interactions with Gly47 and Gly49 of the antibody, respectively. These glycine residues are positioned in close proximity to the CDR-H1, suggesting that this region may be involved in antigen binding. The involvement of residues adjacent to the CDR further highlights the potential importance of local structural flexibility and spatial orientation in stabilizing the antigen–antibody complex. Overall, the docking results provide computational insights into the molecular basis of the D10–A29 interaction, which are consistent with the experimentally observed high affinity of the antibody. However, these predicted interactions are hypothetical and may require experimental validation such as alanine scanning mutagenesis to confirm the actual binding interface.

### 3.5. Specificity Verification and Detection of A29 with D10

**Figure 5:**
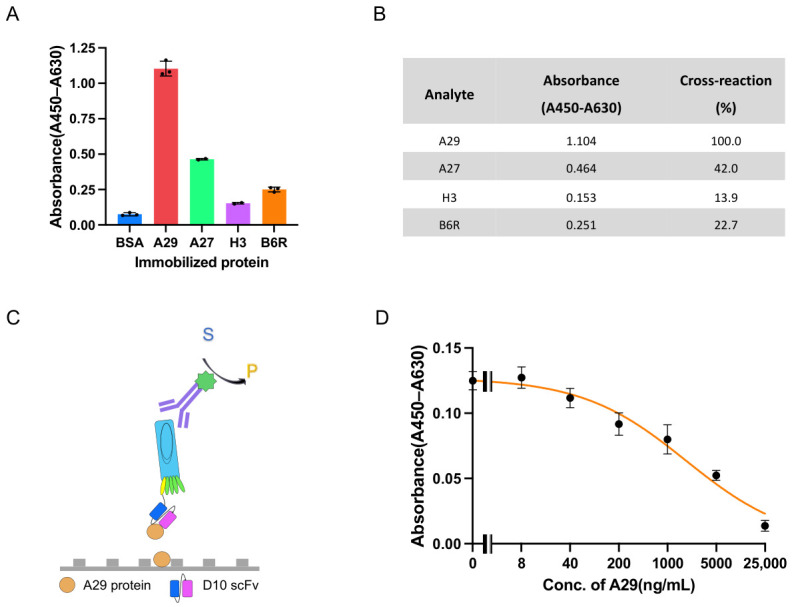
Specificity and quantitative detection performance of the phage-displayed D10 scFv for A29 protein. (**A**) Direct ELISA analysis of D10 scFv binding to immobilized A29, A27, H3, B6R, and BSA. Absorbance values (A450–A630) are shown. (**B**) Cross-reactivity of D10 scFv with A27, H3, and B6R, expressed as a percentage relative to A29 (set as 100%). (**C**) Schematic diagram of the competitive ELISA. Immobilized A29 antigen and free A29 protein compete for binding to the phage-displayed D10 scFv. Higher concentrations of free A29 lead to reduced binding of the antibody to the immobilized antigen, resulting in a decrease in the subsequent absorbance signal. (**D**) Dose–response curve for the quantitative detection of A29 protein. A competitive ELISA was performed using serial concentrations of free A29 protein (0, 8, 40, 200, 1000, 5000, and 25,000 ng/mL). The phage-displayed D10 scFv was pre-incubated with soluble A29 before exposure to the immobilized antigen. The resulting dose–response curve was used to calculate the IC_50_ and determine the LOD for A29 under the established assay conditions. Data are presented as means ± SD (*n* = 3).

The specificity of the D10 scFv was evaluated by a direct ELISA against a panel of immobilized monkeypox virus-related antigens (BSA, A29, A27, H3, and B6R). As shown in Figure 5A, the D10 scFv exhibited the highest binding signal to A29, with negligible reactivity to BSA. Quantitative analysis (Figure 5B) revealed that the cross-reactivity of D10 with A27, H3, and B6R was 42.0%, 13.9%, and 22.7%, respectively. These results indicate that while D10 scFv shows a degree of cross-reactivity with other viral proteins, its strongest and most specific recognition is directed toward A29. Having established its binding specificity, we next evaluated the ability of the phage-displayed D10 scFv to detect free A29 protein in solution using a competitive ELISA. In this assay format (Figure 5C), immobilized A29 antigen and free A29 protein compete for binding to the phage-displayed D10 scFv; the antibody was incubated with varying concentrations of soluble A29 prior to exposure to A29-coated plates. At low concentrations of free A29, minimal competition occurred, and the antibody predominantly bound to the immobilized antigen, resulting in a strong absorbance signal. As the concentration of soluble A29 increased, competition became more pronounced, leading to a progressive reduction in signal intensity. The dose–response curve (Figure 5D) was generated based on the inhibition data, enabling quantitative analysis of antibody performance. The IC_50_ of D10 was calculated to be 1.88 μg/mL, indicating moderate to high binding sensitivity in the competitive format. Furthermore, the LOD for A29 under the established assay conditions was determined to be 0.12 μg/mL, reflecting the assay’s capability to detect low levels of antigen in solution. Taken together, these results demonstrate that the phage-displayed D10 scFv exhibits relatively high specificity and reliable detection performance toward A29, supporting its potential application in immunodiagnostic assays for monkeypox virus detection.

## 4. Discussion

In this study, a novel monoclonal antibody, D10, targeting the A29 protein of MPXV was successfully identified using phage display technology. Its binding activity, affinity, and specificity were systematically characterized. The results demonstrated that D10 exhibits nanomolar-level binding affinity (*K*_D_ = 6.44 nM) and high specificity, with a limit of detection of 0.12 μg/mL in a competitive ELISA format. These findings indicate that D10 performs robustly in the immunodetection of A29 and represents a reliable recognition molecule for the development of rapid diagnostic assays for MPXV. Importantly, this study establishes an efficient strategy for the rapid panning of high-affinity antibodies against emerging viral targets, highlighting its methodological innovation.

Notably, D10 was derived from a human antibody phage display library, which confers significant advantages over conventional hybridoma-derived antibodies [14,15]. The human-origin framework regions of D10 reduce potential immunogenicity and provide a favorable structural basis for downstream applications, including therapeutic development and in vivo diagnostics [13]. In contrast, traditional murine monoclonal antibodies often require complex humanization procedures that may compromise binding affinity and stability [16]. Therefore, the direct isolation of a fully human antibody with high affinity represents a key innovation and translational advantage of this study.

In terms of binding performance, the affinity of D10 falls within the nanomolar range, indicating strong antigen–antibody interactions. Although differences in experimental platforms should be considered, comparison with previously reported antibodies, such as MXV14 (5.82 nM) and MXV15 (13.4 nM) [17], suggests that D10 exhibits competitive binding strength. This demonstrates that the phage display-based panning strategy employed here is capable of efficiently enriching high-affinity antibody candidates.

Regarding detection performance, the competitive ELISA developed in this study achieved an LOD of 0.12 μg/mL for A29. As shown in Figure 5D, the dose–response curve exhibited a clear concentration-dependent inhibition, with an IC_50_ of 1.88 μg/mL, demonstrating that D10 can effectively detect soluble A29. While this sensitivity is lower than that of some recently reported advanced biosensing technologies, such as fiber-optic biolayer interferometry systems capable of sub-ng/mL detection [18], the limitation primarily reflects the intrinsic nature of the competitive ELISA format rather than the binding capability of D10. The competitive format typically yields a higher LOD because it relies on competition between immobilized and free antigen, which reduces the maximal signal. Nevertheless, this format is valuable for applications where the antibody needs to recognize native antigen in solution, such as in clinical sample screening. Future work will focus on incorporating D10 into more sensitive platforms to enhance analytical performance while maintaining specificity.

Molecular docking analysis provided structural insights into the potential antigen–antibody interaction mechanism. The results suggest that D10 may interact with A29 primarily through hydrogen bonding, involving residues Gly47 and Gly49 in the V_H_ region and Arg74 and Arg81 on A29. These residues are located in proximity to the CDR-H1 region, which may contribute to antigen recognition. This structural hypothesis offers a possible explanation for the observed binding affinity. However, these computational predictions are inherently speculative and require experimental validation to confirm the actual binding interface.

D10 showed selective binding to A29 under the tested conditions, including both *E. coli*- and HEK293-derived A29. Although direct ELISA revealed measurable cross-reactivity with A27 (42.0%), B6R (22.7%), and H3 (13.9%) relative to A29, the preferential binding to A29 and the competitive ELISA results indicate that D10 retains acceptable specificity for A29 detection. Additionally, the CDR-H2 sequence of D10 contains an Asp-Gly (DG) motif, which is a known hotspot for succinimide formation and isoAsp generation under acidic or thermal stress. Although no stability studies were performed here, this motif could be optimized by site-directed mutagenesis if enhanced stability is required for long-term storage or diagnostic kit development. Despite these limitations, the combination of high affinity, human origin, and promising detection performance in the competitive ELISA supports the potential utility of D10 in immunodiagnostic platforms such as ELISA and immunochromatographic assays, pending further validation [19,20,21].

Although D10 showed promising performance in vitro, its detection capability in complex biological samples remains to be validated. Furthermore, given the potential sequence variability of the A29 protein among different MPXV strains [22], the breadth of D10 recognition requires further investigation.

## 5. Conclusions

In conclusion, this study successfully obtained a monoclonal antibody, D10, targeting the A29 protein of the monkeypox virus. D10 demonstrates advantages in terms of affinity, specificity, and humanization potential, laying a material foundation for the immunological diagnosis of monkeypox virus and providing a candidate molecule for the development of subsequent therapeutic antibodies.

## Figures and Tables

**Table 1:** Primers used in this study.

Primer Name	Sequence (5′ to 3′)	Length (bp)
AgeI-VH-F	GTGGAACCGGTGAGGTGCAGCTGTTGGAG	30
XhoI-VH-R	AGCGCTCGAGACGGTGACCAGGGTTCCCT	29
SpeI-VL-F	CTAGACTAGTGACATCCAGATGACCCAGT	28
HindIII-VL-R	CCCAAGCTTCCGTTTGATTTCCACCTTGG	28

**Table 2:** The amino acid sequences of V_H_, V_L_ and the CDRs of monoclonal antibody D10.

Sequence Name	Amino Acid Sequences
V_H_	EVQLLESGGGLVQPGGSLRLSCAASGFTFSSYAMSWVRQAPGKGL EWVSTIYGDGSNTYYADSVKGRFTISRDNSKNTLYLQMNSLRAED TAVYYCAKNYSTFDYWGQGTLVTVSS
CDR-H1	GFTFSSYA
CDR-H2	IYGDGSNT
CDR-H3	AKNYSTFDY
V_L_	TDIQMTQSPSSLSASVGDRVTITCRASQSISSYLNWYQQ KPGKAPKLLIYTASNLQSGVPSRFSGSGSGTDFTLTISSL QPEDFATYYCQQGASAPATFGQGTKVEIKRAAA
CDR-L1	QSISSY
CDR-L2	TASN
CDR-L3	QQGASAPAT

**Table 3:** Kinetic parameters of D10 Fab for A29-binding.

	*K*_on_ (1/Ms) × 10^4^	*K*_dis_(1/s) × 10^−4^	*K*_D_ (nM)	*R* ^2^
D10 Fab	3.32 ± 0.03	2.21 ± 0.12	6.44	0.94

## Data Availability

The original contributions presented in this study are included in the article. Further inquiries can be directed to the corresponding author.

## Abbreviations

The following abbreviations are used in this manuscript:

MPXV	Monkeypox Virus
COVID-19	Corona Virus Disease 2019
IMV	Intracellular Mature Virion
PCR	Polymerase Chain Reaction
K_D_ value	Dissociation Equilibrium Constant
CDR	Complementarity Determining Region
BLI	Biolayer Interferometry
ELISA	Enzyme-linked Immunosorbent Assay
SDS-PAGE	Sodium Dodecyl Sulfate-polyacrylamide Gel Electrophoresis
IC_50_	Half-inhibitory Concentration
LOD	Limit of Detection
pLDDT	Predicted Local Distance Difference Test
TMB	Tetramethylbenzidine
BSA	Bovine Serum Albumin
HRP	Horseradish Peroxidase
Fab	Antigen-binding fragment
scFv	Single-chain variable fragment
SA	Streptavidin

## References

[1] Islam M.A., Mumin J., Haque M.M., Haque M.A., Khan A., Bhattacharya P., Haque M.A. (2023). Monkeypox virus (MPXV): A Brief account of global spread, epidemiology, virology, clinical features, pathogenesis, and therapeutic interventions. Infect. Med..

[2] Kumari R., Arya P., Yadav S.P., Mishra R.C., Yadav J.P. (2024). Monkeypox Virus (MPXV) Infection: A Review. Infect. Disord. Drug Targets.

[3] Khan A., Erickson T.A., Carrillo L. (2025). COVID-19 and MPXV: Twindemic Response and Dual Infections in Individuals in a US Metro. Epidemiologia.

[4] Harapan H., Ophinni Y., Megawati D., Frediansyah A., Mamada S.S., Salampe M., Bin Emran T., Winardi W., Fathima R., Sirinam S. (2022). Monkeypox: A Comprehensive Review. Viruses.

[5] Liang C.Y., Chao T.L., Chao C.S., Liu W.D., Cheng Y.C., Chang S.Y., Chang S.C. (2024). Monkeypox virus A29L protein as the target for specific diagnosis and serological analysis. Appl. Microbiol. Biotechnol..

[6] Hughes L.J., Goldstein J., Pohl J., Hooper J.W., Lee Pitts R., Townsend M.B., Bagarozzi D., Damon I.K., Karem K.L. (2014). A highly specific monoclonal antibody against monkeypox virus detects the heparin binding domain of A27. Virology.

[7] Absil G., Sougne L., Lahrichi D., Collins P., Meuris C., Moutschen M., Nikkels A.F., Orban C. (2022). [Monkeypox]. Rev. Med. Liege.

[8] Khoo Y.W., Li S., Chong K.P. (2022). In-silico primer designing and PCR for detection of monkeypox virus (MPXV). J. Infect. Public Health.

[9] Wang W., Li J.X., Long S.Q., Liu Z.N., Li X.P., Peng Z.H., Zheng J.D., Liao Y.H. (2025). Emerging strategies for monkeypox: Antigen and antibody applications in diagnostics, vaccines, and treatments. Mil. Med. Res..

[10] Davis I., Payne J.M., Olguin V.L., Sanders M.P., Clements T., Stefan C.P., Williams J.A., Hooper J.W., Huggins J.W., Mucker E.M. (2023). Development of a specific MPXV antigen detection immunodiagnostic assay. Front. Microbiol..

[11] Qu J., Zhang X., Liu K., Li Y., Wang T., Fang Z., Chen C., Tan X., Lin Y., Xu Q. (2024). A Comparative Evaluation of Three Diagnostic Assays for the Detection of Human Monkeypox. Viruses.

[12] Pang S., Wang M., Yuan J., Yang Z., Yu H., Zhang H., Dong T., Liu A. (2024). Sensitive Dual-Signal ELISA Based on Specific Phage-Displayed Double Peptide Probes with Internal Filtering Effect to Assay Monkeypox Virus Antigen. Anal. Chem..

[13] Frenzel A., Schirrmann T., Hust M. (2016). Phage display-derived human antibodies in clinical development and therapy. mAbs.

[14] Ossysek K., Uchański T., Kulesza M., Bzowska M., Klaus T., Woś K., Madej M., Bereta J. (2015). A new expression vector facilitating production and functional analysis of scFv antibody fragments selected from Tomlinson I+J phagemid libraries. Immunol. Lett..

[15] Saw P.E., Song E.W. (2019). Phage display screening of therapeutic peptide for cancer targeting and therapy. Protein Cell.

[16] Yoshikawa K., Ueda R., Obata Y., Utsumi K.R., Notake K., Takahashi T. (1986). Human monoclonal antibody reactive to stomach cancer produced by mouse-human hybridoma technique. Jpn. J. Cancer Res..

[17] Yan H., Su J., Tian L., Li Q., Feng X., Zhang J., Shi Y., Liao C., Liu J., Gao S. (2024). A rapid and sensitive fluorescent chromatography with cloud system for MPXV point-of-care diagnosis. Anal. Chim. Acta.

[18] Song X., Tao Y., Bian S., Sawan M. (2024). Optical biosensing of monkeypox virus using novel recombinant silica-binding proteins for site-directed antibody immobilization. J. Pharm. Anal..

[19] Lee C.H., Wu C.J., Chiang J.Y., Yen F.Y., Shen T.J., Leu S.J., Tsai B.Y., Mao Y.C., Andriani V., Wang W.C. (2025). Chicken-Derived Single-Chain Variable Fragments Targeting Monkeypox Virus A29L Protein. Biotechnol. J..

[20] Li L., Jia Q., Lai H., Luo G., Lu Y., Liang H., Dong W., Chen C. (2025). Development of a one-step diagnostic and differential immunochromatographic method for mpox and chickenpox. J. Immunol. Methods.

[21] Ye L., Lei X., Xu X., Xu L., Kuang H., Xu C. (2023). Gold-based paper for antigen detection of monkeypox virus. Analyst.

[22] Sun X., Zhang L., Chen G., Yang F., Ning X., Qiu J., Gao Y., Yang J., Zhang W., Zhang Z. (2026). A cocktail vaccine with monkeypox virus antigens confers protection without selecting mutations in potential immune evasion genes in the vaccinia WR strain challenge. mBio.

